# Ceruloplasmin Is a Potential Biomarker for aGvHD following Allogeneic Hematopoietic Stem Cell Transplantation

**DOI:** 10.1371/journal.pone.0058735

**Published:** 2013-03-07

**Authors:** Meng Lv, Hai-ge Ye, Xiao-su Zhao, Xiang-yu Zhao, Ying-jun Chang, Dai-hong Liu, Lan-ping Xu, Xiao-jun Huang

**Affiliations:** 1 Peking University People's Hospital, Peking University Institute of Hematology, Beijing Key Laboratory of Hematopoietic Stem Cell Transplantation, Beijing, China; 2 Department of Hematology, The First Affiliated Hospital of Wenzhou Medical College, Zhejiang, China; West Virginia University School of Medicine, United States of America

## Abstract

Acute graft-versus-host-disease (aGvHD) is the major cause of non-relapse mortality after allogeneic hematopoietic stem cell transplantation (allo-HSCT). Recently, diagnostic biomarkers for aGvHD have been shown to play important roles in evaluating disease status and mortality risk after allo-HSCT. To identify plasma biomarkers for aGvHD with high sensitivity and specificity, a quantitative proteomic approach using 8-plex isobaric tags for relative and absolute quantitation (8-plex iTRAQ) was employed to screen differentially expressed proteins in peripheral blood before and after the onset of aGvHD. Four target proteins, ceruloplasmin (CP), myeloperoxidase (MPO), complement factor H (CFH), and alpha-1-acid glycoprotein (AGP), were chosen for preliminary validation with enzyme linked immunosorbent assay (ELISA) in 20 paired samples at both the time of diagnosis of aGvHD and the time of complete response. The most promising candidate, ceruloplasmin, was further validated at fixed time points after allo-HSCT and during aGvHD. The plasma ceruloplasmin levels were significantly increased during the period of aGvHD onset and were markedly decreased as aGvHD resolved. The plasma ceruloplasmin levels at different time points post-transplant in the aGvHD (+) group were significantly higher than those in the aGvHD (−) group (p<0.001). The elevation of ceruloplasmin level in patients with active aGvHD was independent of infection status. Patients whose ceruloplasmin levels were elevated above 670 μg/ml at 7, 14 and 21 days after allo-HSCT had a remarkably increased probability of subsequently developing aGvHD. In conclusion, our results suggest that plasma ceruloplasmin is a potential plasma biomarker of aGvHD, and it also has prognostic value for risk-adapted prophylaxis during the consecutive time points monitored in the first month after allo-HSCT.

## Introduction

Acute graft-versus-host-disease (aGvHD), one of the major causes of non-relapse mortality after allogeneic hematopoietic stem cell transplantation (allo-HSCT), involves damage to target organs by alloreactive T cells and manifests as alterations of the skin, gastrointestinal tract and liver function [1]. The diagnosis of aGvHD is based on clinical criteria and biopsy results of the involved organs, which fail to maintain a balance of accuracy and convenience. In recent years, deeper insights into the complex pathophysiology of aGvHD have enabled the development of the levels of associated proteins in the peripheral blood of patients after allo-HSCT as useful biomarkers for the diagnosis of aGvHD [2]–[5]. Additionally, using a combination of biomarkers in such panels further improves their diagnostic value. Levine, et al. reported that in a phase 2 clinical trial, a 6-protein biomarker panel consisting of IL-2 receptor-α, tumor necrosis factor receptor-1, hepatocyte growth factor, IL-8, elafin and regenerating islet-derived 3-α could predict post-therapy nonresponse and mortality [6]. Similar to the use of well-established tumor biomarkers for the management of certain cancers, measurements of aGvHD biomarker concentrations in multi-marker panels could be incorporated into routine clinical follow-up. Therefore, it is important to enrich the pool of putative biomarkers to incorporate into aGvHD biomarker panels that might further improve the specificity and/or sensitivity of diagnosis.

Recently, we reported the utilization of 8-plex isobaric tags for relative and absolute quantitation (8-plex iTRAQ), a quantitative proteomic technique, to screen for aGvHD biomarkers in an unbiased fashion without considering the availability of antibodies for protein chips. The use of 8-plex iTRAQ coupled with strong cation exchange (SCX) and liquid chromatography-tandem mass spectrometry (LC-MS/MS) increased the throughput of the analysis while reducing experimental error, which allowed the identification of potential biomarkers related to immunity or tissue damage [7]. One candidate, lipopolysaccharide-binding protein (LBP), was discovered through the use of 8-plex iTRAQ and was validated for the diagnosis and prediction of aGvHD development [8].

Here, we report the discovery and validation of a new candidate plasma biomarker for aGvHD, ceruloplasmin (CP), a 151-kDa protein that controls iron metabolism hemostasis and is well known for its diagnostic value in Wilson's disease [9]. Ceruloplasmin has also been reported to be an acute-phase protein related to various acute inflammatory conditions, including injury, tumors, and cardiovascular disease [10], [11]. The role of ceruloplasmin as an aGvHD biomarker after allo-HSCT had not been previously investigated.

## Materials and Methods

### Ethics Statement

This study was approved by the Ethics Committee of Peking University People's Hospital, Beijing in accordance with the Declaration of Helsinki. Written form of informed consent was obtained from participants with full civil capability (age>18 year), or from parents/guardians of minors participants with limited civil capability (age 16–18 year).

### Patient Enrollment

Patients who had undergone either unmanipulated HLA-matched or haploidentical allogeneic blood and marrow transplantation from related donors or HLA-matched unrelated allogeneic blood transplantation at the Peking University People's Hospital between June 2010 and June 2011 were enrolled in this study. The enrollment criteria included adult patients (age 16–50 year) with hematological malignancies, including leukemia, lymphoma, and myelodysplastic syndrome (MDS). Participants were assigned, based on their own choice, to either systemic monitoring after allo-HSCT or symptomatic follow-up after aGvHD occurrence. Additional patients within 100 days after allo-HSCT were enrolled for symptomatic follow-up for infections in Dec 2012.

### Transplant procedures and aGvHD Prophylaxis

The transplant procedures used have been previously described [12]–[14]. Briefly, 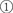 the recipients of HLA-identical related transplants received cytosine arabinoside (2 g/m^2^ for 2 days), busulfan (0.8 mg/kg IV every 6 hours, for a total of 12 doses), cyclophosphamide (1.8 g/m^2^/d IV for 2 days), and semustine (250 mg/m^2^ PO for 1 day). Recipients of HLA-haploidentical related transplants and recipients of HLA-matched transplants from unrelated donors were conditioned with cytosine arabinoside (4 g/m^2^ for 2 days), busulfan (0.8 mg/kg IV every 6 hours, for a total of 12 doses), cyclophosphamide (1.8 g/m^2^/d IV for 2 days), semustine (250 mg/m^2^ PO for 1 day), and human anti-thymocyte globulin (2.5 mg/kg/d IV for 4 days) (SangStat/Genzyme). 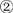 Grafts were transfused that combined G-CSF-mobilized bone marrow and peripheral blood harvest in related transplantation patients or G-CSF-mobilized peripheral blood harvest in unrelated transplantation patients. The day on which graft infusion was defined as day 0, and days before infusion are preceded by “−“, and the days after the last stem cell infusion are preceded by “+”. 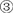 GvHD prophylaxis mainly combined cyclosporine A (CsA), methotrexate (MTX) and mycophenolate mofetil (MMF). CsA was begun intravenously on day^−9^ at a dose of 2.5 mg/kg and was replaced by an oral formulation as soon as the patient was able to tolerate oral medication after engraftment. The dosage was adjusted to maintain the blood levels between 150 and 250 ng/ml and was gradually tapered after 3 months. Oral MMF (1 g daily) was begun on day _9 and was discontinued after engraftment in the HLA-matched sibling transplants. In the unrelated donor transplants, the MMF was tapered from 1 g to 0.5 g daily after engraftment and was discontinued on day +30. In the HLA-mismatched/haploidentical sibling donor transplants, the MMF was tapered from day +30 and was discontinued on day +60. MTX was administered IV at dosages of 15 mg/m^2^ on day 1 and 10 mg/m^2^ on days +3 and +6. For haploidentical transplantation, an additional 10 mg/m^2^ of MTX was administered on day +11.

### aGvHD diagnosis and its management

aGvHD was diagnosed according to the Glucksberg-Seattle criteria, and gastrointestinal (GI-type) aGvHD was confirmed by biopsy with a colonoscopy enteroscope [15]. Complete response to aGvHD therapy (CR) was defined as the complete resolution of aGvHD symptoms in all organs, without secondary GVHD therapy, which included the absence of an erythematous rash attributable to GVHD; a total serum bilirubin concentration <2.0 mg/dL in the absence of hepatic complications other than GVHD or <3.0 mg/dL in the presence of such complications; the absence of anorexia, nausea or vomiting attributable to GVHD; and the presence of at least intermittent formed stools [16], [17].

Patients with aGvHD were treated with 1 mg/kg/day of methylprednisolone equivalent and the resumption of full-dose CsA therapy. Second-line therapy, which primarily consisted of CD25 monoclonal antibody (Simulect, Novartis) or tacrolimus (FK506), MMF, and MTX, was administered for steroid-refractory aGvHD.

### Sample Collection

For patients under systemic monitoring, K2-EDTA-anticoagulated whole blood (2 mL) was routinely collected at the following fixed time points: d-9 (before conditioning), d-1 (one day before graft transfusion), d+7, d+14, d+21, d+28, d+55 to d+60(d60), and d+90 to d+100(d+90).

For patients under systemic monitoring and symptomatic follow-up after aGvHD, blood samples were collected at the time of diagnosis of aGvHD and then twice per week until aGvHD got CR.

For patients symptomatic follow-up for infections, blood samples were collected at the time of diagnosis of infections without aGvHD until the clinical manifestation and serological evidence become negative.

The blood sample was centrifuged at 1500 rpm for 10 min to obtain platelet-poor plasma, which was then stored at −80°C until testing.

### Proteomic Profiling

The 8-plex iTRAQ quantitative proteomic technique, including sample preparation, high-abundance protein removal, buffer exchange, protein concentration assay, protein reduction, alkylation, trypsin digestion, iTRAQ labeling and peptide fractionation with SCX chromatography, was performed as previously described [7].

### ELISA Assay

The samples were diluted, and the target proteins were measured using ELISA kits at the following antibody concentrations, according to each manufacturer's protocol: ceruloplasmin, 1:400 (Assaypro, EC4001-1); myeloperoxidase, 1:20 (Bender, BMS2038INST); complement factor H, 1:20000 (Hycult Biotech, HK342); and alpha-1-acid glycoprotein, 1:2000 (Assaypro, EG5001-1). The samples and standards were assayed in duplicate, the absorbance was measured using a Model 680 microplate reader (Bio-Rad), and the results were calculated with SoftMax Pro 6 (Molecular Devices). The operators of the ELISA assays were blinded to the clinical background of the samples.

### Statistical Analysis

All comparisons were performed using either a parametric t-test or the nonparametric Mann-Whitney U test. The cutoff value for ceruloplasmin was determined using a receiver operating characteristic (ROC) curve. The analysis was performed using SPSS 19.0 statistical software. P value smaller than 0.05 was considered statistically significant.

## Results

### (1) Patient Enrollment and Samples

A total of 98 patients were enrolled in this cohort between June 2010 and June 2011, and samples from 63 patients were available for systemic monitoring from all of the fixed time points: d-9, d-1, d+7, d+14, d+21, d+28, d+56 to d+60 and d+90 to d+100. Of these 63 patients, 37 patients developed aGvHD. The other 35 patients were available at active and complete response of aGvHD (Table-1, Figure S1). Of the total 72 patients with aGvHD, 54 patients had a complete response to corticosteroid treatment, and the 18 steroid-refractory patients received second-line therapy. Additional 7 patients with infections in the absence of aGvHD were enrolled in Dec 2012.

**Table 1:** Characteristics of the patients, donors, and grafts.

	Total	Systemic follow-up	Symptomatic follow-up
Number(No.) of Patients	98	63	35
Median age (Range)	24(16–48)	26(16–47)	23(16–48)
Gender of recipients, Male No. (%)	58(59%)	35(56%)	23(66%)
Diagnosis, No. (%)			
AML	42(42%)	30(48%)	12(34%)
ALL	37(37%)	22(35%)	15(41%)
CML	9 (10%)	7(10%)	2(6%)
MDS	7 (6%)	4(6%)	3(9%)
NHL	3(2%)	0(0%)	3(9%)
Donor match (%)*			
Matched	32(30%)	20(32%)	12(34%)
Mismatched	66(67%)	43(68%)	23(66%)
one locus	4	3	1
two loci	19	10	9
three loci	43	40	13
Source of grafts, no. of patients (%)			
BM+PBSC	96(98%)	63	33
PB	2(2%)	0	2
aGvHD	72	37	35
Time, median days(range)	25(15–68)	24(15–52)	27(16–68)
Grade-1	36	16	20
Grade-2	32	19	13
Grade-3	3	2	1
Grade-4	1	0	1

Abbreviations: aGvHD = acute graft-versus-host disease; AML: acute myeloid leukemia; ALL: acute lymphoblastic leukemia; CML: chronic myelogenous leukemia; MDS: myelodysplastic syndromes; NHL: Non Hodgkin lymphoma.

* Related donors were required to match the recipients for the serological defined HLA-A and-B antigens as well as HLA-DRB1 alleles. HLA-A and -B antigens were typed by DNA methods and HLA-DRB1 alleles were typed with sequence-specific oligonucleotide probes. One locus mismatch meant 5/6 and two loci mismatch meant 4/6. Unrelated donors were additionally required to match HLA-C and HLA-DQB1 alleles.

### (2) Proteomic screening and preliminary validation

**Figure 1:**
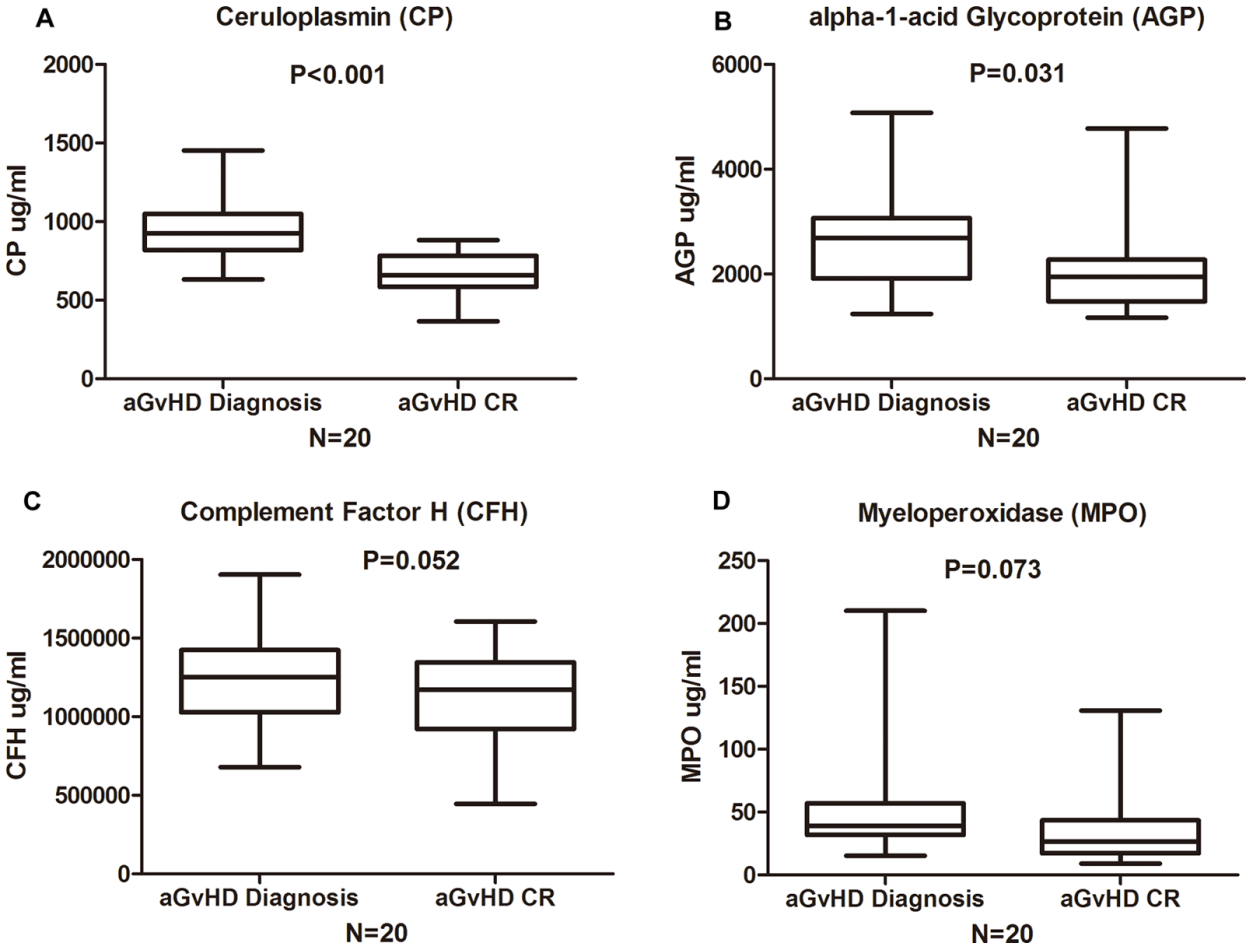
Preliminary validation of four candidate proteins. Four candidate proteins underwent preliminary validation were tested by ELISA in paired samples from 20 patients at active aGvHD onset and after complete response(CR). (A) Ceruloplasmin (CP) (Diagnosis Vs. CR p<0.001), (B) alpha-1-acid glycoprotein (AGP) (Diagnosis Vs. CR p = 0.031), (C) Complement factor H (CFH) (Diagnosis Vs. CR p  = 0.052), (D) Myeloperoxidase (MPO) (Diagnosis Vs. CR p = 0.073).

The 8-iTRAQ quantitative proteomic technology was applied to plasma samples from 4 patients with biopsy-proven aGvHD who had a complete response to therapy (1 grade 1 skin type; 2 grade 2 GI type; and 1 grade 2 mixed type cases) without infections. Twenty-one proteins were up-regulated more than 1.2-fold at diagnosis compared with after complete response. Of these 21 proteins, 4 that had not been studied for their clinical correlation with aGvHD were chosen as candidates (Table S1): ceruloplasmin (CP), myeloperoxidase (MPO), complement factor H (CFH) and alpha-1-acid glycoprotein (AGP). These four proteins underwent preliminary validation by ELISA in paired samples from another 20 patients (at aGvHD diagnosis and after complete response). The plasma levels of CP (p<0.001) and AGP (p = 0.031) were significantly higher at diagnosis, whereas there were no differences between the diagnosis and complete response levels of MPO (p = 0.073) and CFH (p = 0.052), as shown in Figure-1. In addition, 100% of the patients' CP levels were higher during the active aGvHD phase, whereas 25% of the patients' AGP levels were higher after complete response; thus, ceruloplasmin was chosen as the candidate biomarker for further validation.

### (3) Kinetics of ceruloplasmin in patients after allo-HSCT

**Figure 2:**
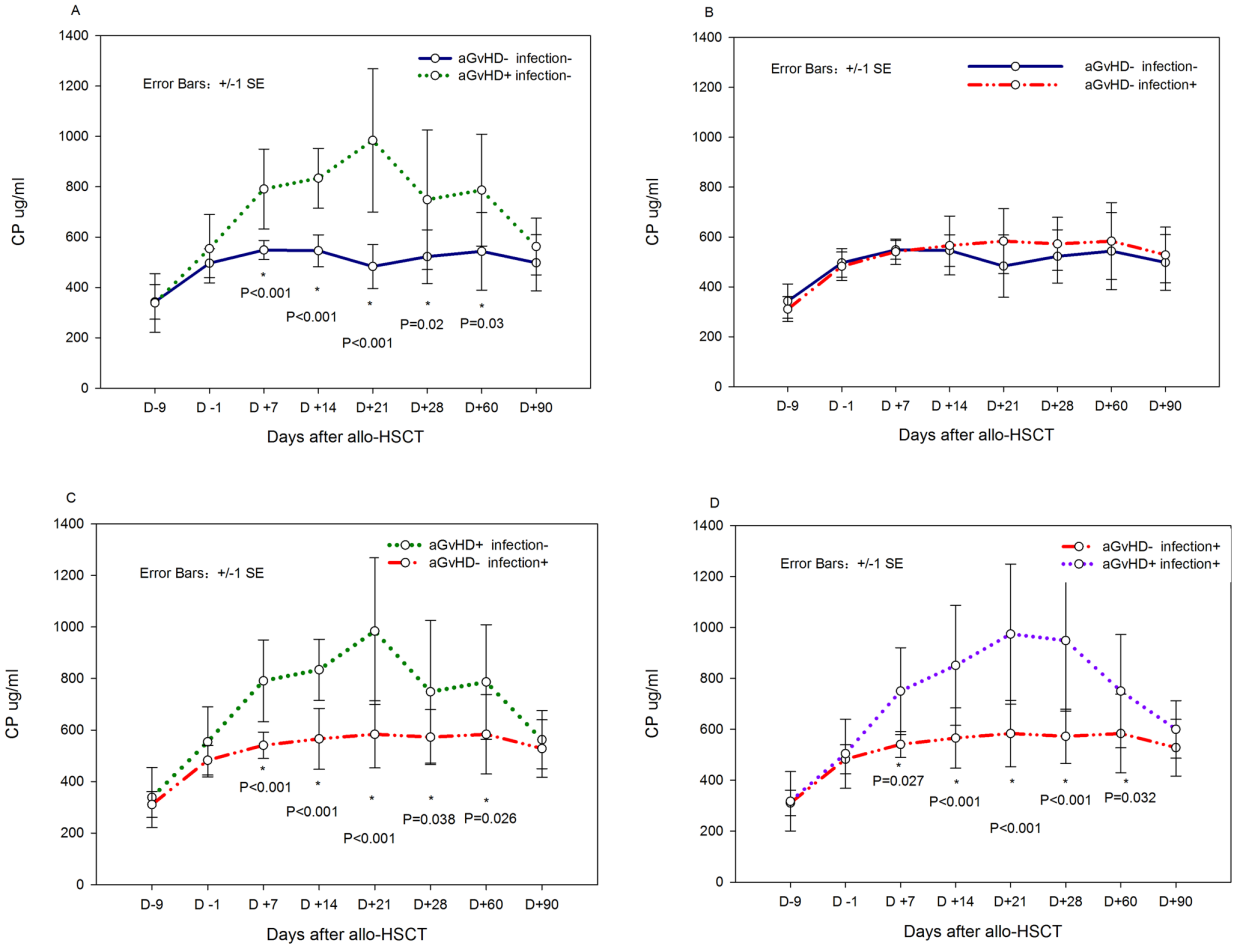
Kinetics of ceruloplasmin following allo-HSCT. (A) comparison of 16 aGvHD+infection- and 15 aGvHD-infection- patients. (B) comparison of 11 aGvHD-infection+ and 15 aGvHD-infection- patients. (C) comparison of 16 aGvHD+infection- and 11 aGvHD-infection+ patients. (D) comparison of 21 aGvHD+infection+ and 11 aGvHD-infection+ patients.

Because ceruloplasmin is an acute-phase reactant that may be involved in inflammatory processes such as infection, 31 patients without infections at fixed time points were chosen for the evaluation of the impact of only aGvHD on the kinetics of ceruloplasmin following allo-HSCT. The characteristics of 16 patients with aGvHD (aGvHD+) and another 15 without aGvHD (aGvHD-) are summarized in Table-2. The ceruloplasmin levels of the aGvHD+ patients from d+7 to d+56 were significantly higher than those of the aGvHD- patients (Figure-2A). The ceruloplasmin levels of the two groups rose from the time of conditioning (d-9) to the graft infusion (d-1) (p<0.001). However, only the ceruloplasmin levels of the aGvHD+ group continued to increase from d-1 to d+7 (p<0.001); the aGvHD- group exhibited no significant changes (P = 0.060).

**Table 2:** Patients Characteristics of aGVHD+ and aGVHD- groups in kinetic study.

	aGVHD+ group	aGVHD- group	
No. of patients (male/female)	16(9/7)	15(9/6)	P Value
Age of recipients: median (range),years	25(17–45)	26(16–43)	N.S
Infused MNC (x10^8^/kg): median (range)	8.1(4.2–12.1)	7.9(4.7–11.2)	N.S
All CD3+ T cells	1.8(0.8–5.2)	1.7(0.9–4.9)	N.S
CD4+ T cells	1.0(0.4–2.9)	1.0(0.5–2.5)	N.S
CD8+ T cells	0.8(0.3–2.5)	0.7(0.3–2.3)	N.S
Donor match, no:			
Mismatched, related	11	9	N.S
Matched, sibling	5	6	N.S
CP value (ng/ml): median(SD)			
Day -9	338 (116.58)	343(69.01)	N.S
Day -1	550(91.275)	496(57.14)	N.S
Day+7	650(124.93)	549(37.92)	0.001
Day+14	879(244.76)	546(63.18)	<0.001
Day+21	935(242.11)	483(87.19)	<0.001
Day+28	668(195.95)	523(106.46)	0.001
Day+56	714(218.50)	544(154.34)	0.003
Day+90	502(68.34)	498(111.45)	N.S

Abbreviations: NS, Not Statistically Significant. SD: Standard Deviation.

Then a cohort of patients were analyzed for the influence of only infections on the kinetics of ceruloplasmin following allo-HSCT, we compared the ceruloplasmin levels of 11 infections+aGvHD- and another 15 infection-aGvHD- patients at multi-time points, there was no difference between these two groups (Figure-2B)

It was also found ceruloplasmin level of aGvHD+infection- patients (n = 16) were significantly higher than aGvHD-infection+ patients (n = 11) from d+7 to d+60 (Figure-2C).Similar patterns were found in aGvHD+infection+ patients (n = 21) and aGvHD-infection+ patients (N = 11) (Figure-2D).

### (4) The diagnostic value of ceruloplasmin in aGvHD

**Figure 3:**
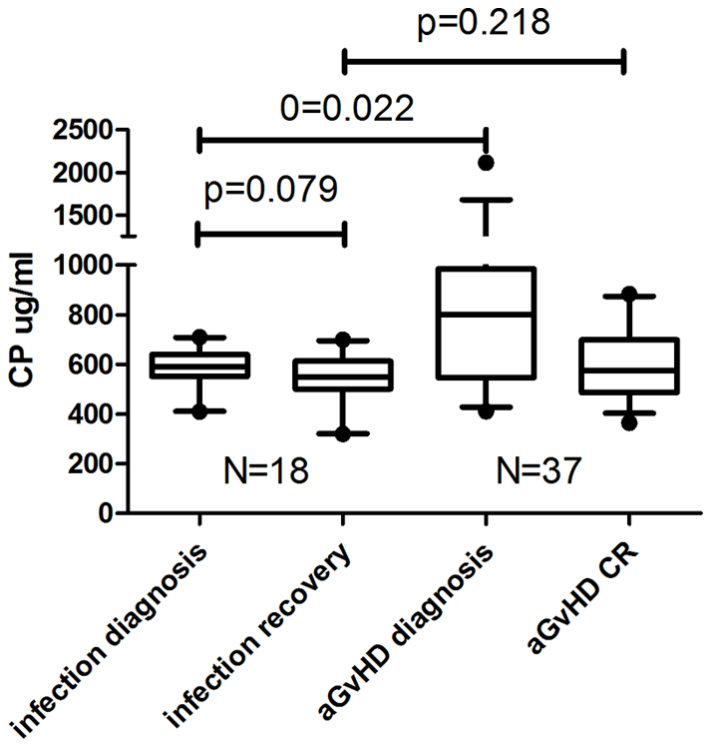
Comparison of ceruloplasmin levels in infection and aGvHD. Infection+aGvHD- (n = 18; systemic n = 11; symptomatic n = 7) and aGvHD+infection- (n = 37; systemic n = 16; symptomatic n = 21) were compared at diagnosis of complications as well as recovery.

To clarify the potential meaning of ceruloplasmin in the differential diagnosis of aGvHD and infection, patients of infection+aGvHD- (n = 18; systemic n = 11; symptomatic n = 7) and aGvHD+infection-(n = 37; systemic n = 16; symptomatic n = 21) were compared at diagnosis of complications as well as recovery. The ceruloplasmin levels at diagnosis of aGvHD (infection-) were higher than infection (aGvHD-) (p = 0.022), while there were no differences between aGvHD and infection groups as patients got recovery (p = 0.218) . (Figure-3).

What's more, in the total 72 cases of aGvHD in systemic monitoring as well as symptomatic follow-up, infection+ and infection- patients had similar ceruloplasmin level at diagnosis of different grades of aGvHD. (grade1, p = 0.972 and grade-2, p = 0.436; respectively) (Figure S2).

**Figure 4:**
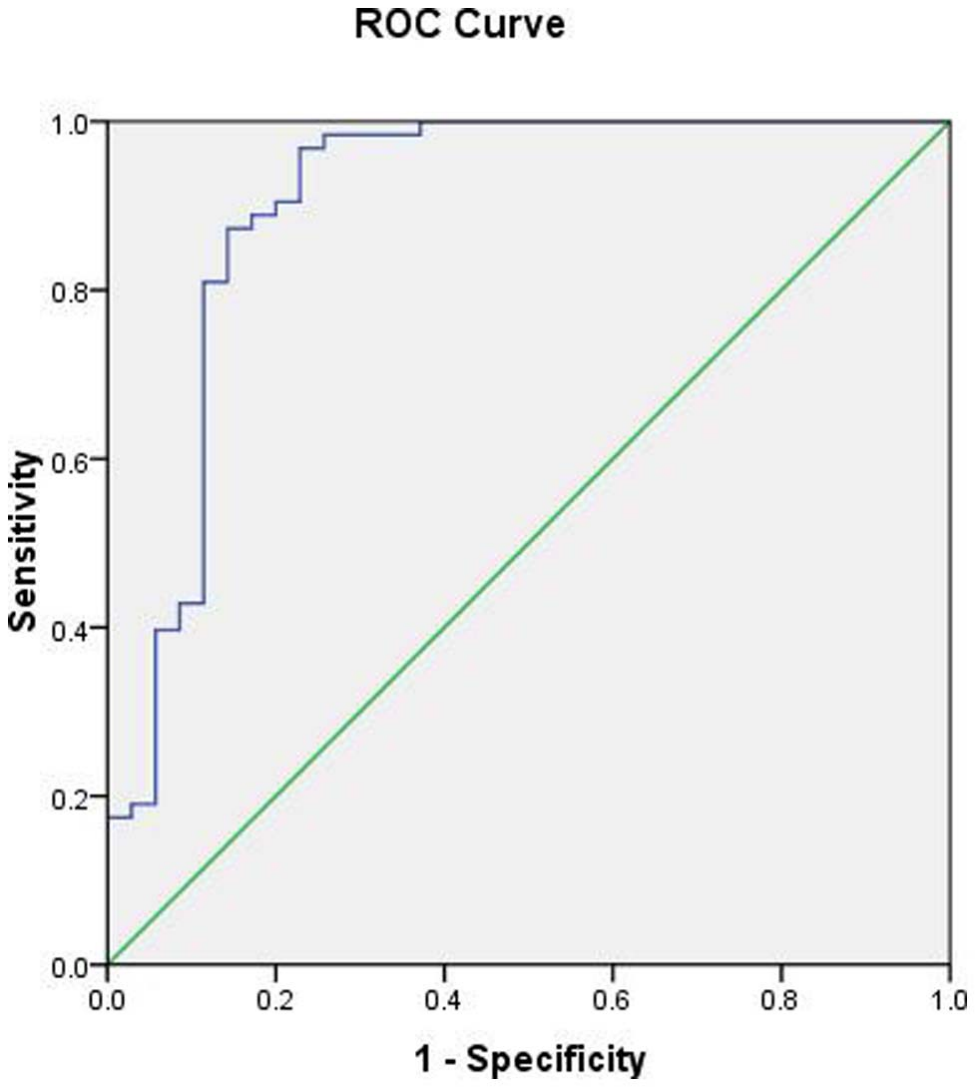
ROC analysis of ceruloplasmin levels at aGvHD diagnosis. (ROC) curve analysis of patients with aGvHD (n = 72) and without aGvHD (n = 26). At a cutoff value of 780 μg/ml, the corresponding sensitivity was 0.905, the specificity was 0.8, the area under the ROC was 0.902 (95% CI 0.825, 0.979; P<0.001), and the standard error was 0.039.

To determine the cutoff value of ceruloplasmin for the accurate and specific diagnosis of aGvHD, we performed a receiver operating characteristic (ROC) curve analysis of patients with aGvHD (n = 72) and without aGvHD (n = 26). At a cutoff value of 780 μg/ml, the corresponding sensitivity was 0.905, the specificity was 0.8, the area under the ROC was 0.902 (95% CI 0.825, 0.979; P<0.001), and the standard error was 0.039. (Figure-4).

### (5) The relationship between ceruloplasmin levels and aGvHD characteristics

**Figure 5:**
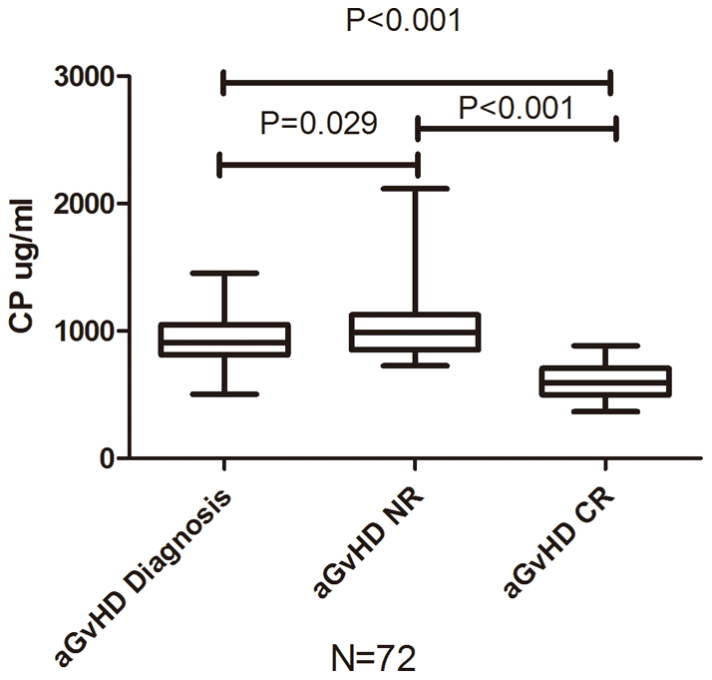
Ceruloplasmin levels during aGvHD recovery. Comparison of ceruloplasmin level in patients with aGvHD (n = 72) at diagnosis, none remission (NR), complete response (CR).

As shown in preliminary validation, the ceruloplasmin levels increased significantly during active aGvHD (N = 20, p<0.001). We further validated the CP levels in 72 patients over the course of aGvHD. The ceruloplasmin levels continued to increase in patients with non-remission (NR) or worsened aGvHD (P = 0.029). However, the levels decreased as the aGvHD got complete response (P<0.001) (Figure-5). While the median ceruloplasmin level of the grade 2 aGvHD patients was higher than that of the grade 1 aGvHD patients (1073 vs. 942 μg/ml), the two groups were not statistically different (P = 0.206). Nevertheless, the level of ceruloplasmin at diagnosis failed to predict which patients would be resistant to the first-line anti-aGvHD therapy (n = 18).

**Figure 6:**
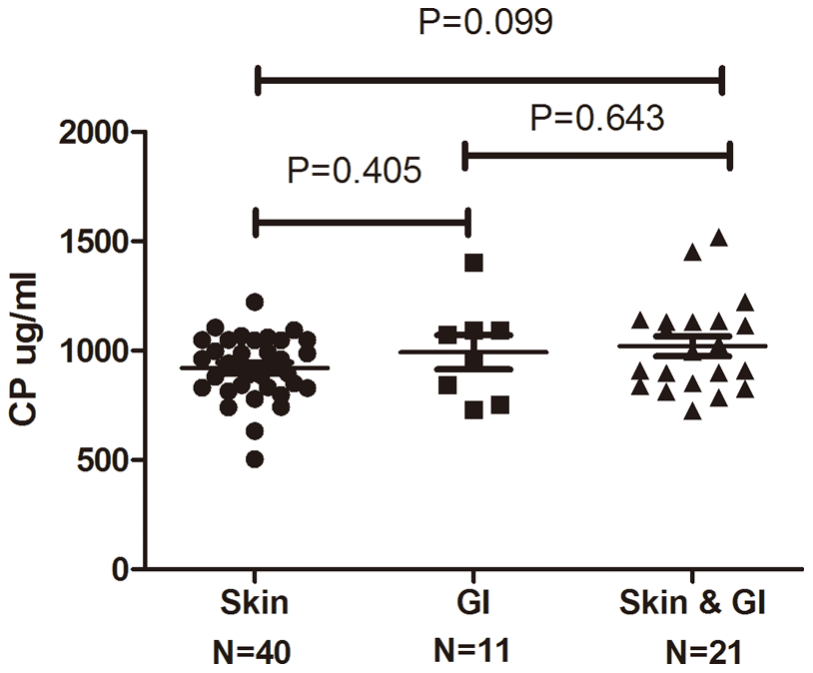
Ceruloplasmin levels in different types of aGvHD. Comparison of ceruloplasmin level in patients with different aGvHD involved organs: Skin (n = 40), Gastrointestinal (GI, n = 11) and Mixed (Skin & GI, n = 21).

Furthermore, there was no organ specificity of the ceruloplasmin level at the time of aGvHD diagnosis. The ceruloplasmin levels of the mixed-type aGvHD patients tended to be higher than those of the skin-type aGvHD patients, but the difference was not statistically significant (P = 0.099) (Figure-6).

In addition, patients who had received allo-HSCT from matched related donors ( MRD,n = 20) and haplo-identical donors (Haplo, n = 43) in systemic following-up were compared at multi-time points, there were not significant differences between these two groups in ceruloplasmin (Figure S3).

### (6) The prognostic value of ceruloplasmin for aGvHD during the first month after allo-HSCT

As shown in the ceruloplasmin kinetics study, the ceruloplasmin levels of the aGvHD+ versus the aGvHD- patients exhibited differences between d+7 and d+56. Therefore, it is possible to predict aGvHD onset before clinical symptoms develop by evaluating ceruloplasmin at fixed time points after HSCT, to identify the period of “aGvHD alarming”.

**Figure 7:**
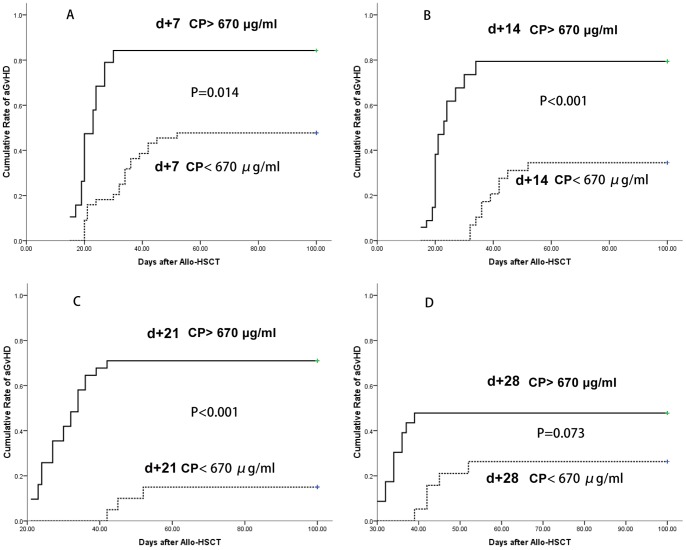
The cumulative incidence of aGvHD at multiple time points. Comparison of cumulative incidence of aGvHD in two groups of patients (CP> 670 μg/ml or <670 μg/ml) at different time points: d+7(p = 0.014), d+14(p<0.001), d+21(p<0.001) and d+28(p = 0.073).

As all aGvHD occurred before d+56 in patients undergoing systemic monitoring, the cutoff value of ceruloplasmin for prognosis was determined in samples between d+7 to d+28. ROC statistics were performed, and 670 μg/ml was chosen as the cutoff value: at this level, the sensitivity was 79.7% and the specificity was 78.3%. The area under the ROC was 0.861 (95% CI 0.795, 0.926; P = 0.000), and the standard error was 0.033. The cumulative incidence of aGvHD was analyzed to compare two groups of patients undergoing systemic monitoring (CP> 670 μg/ml or <670 μg/ml )at different time points (d+7, d+14, d+21 and d+28). The results indicated that the probability of developing aGvHD was significantly different for patients with ceruloplasmin levels above or below 670 μg/ml at 3 time points (d+7, d+14 and d+21) (Figure-7).

## Discussion

Our previous study established the utility of 8-plex iTRAQ quantitative proteomic technology for aGvHD biomarker screening and identified LBP as a candidate aGvHD biomarker. In the current cohort study, we further investigated several newly found candidates, ceruloplasmin, myeloperoxidase, complement factor H and alpha-1-acid glycoprotein, by comparing their plasma levels during the active phase of aGvHD and after the complete response to aGvHD therapy. For the first time, we validated ceruloplasmin as a potential biomarker for aGvHD diagnosis and risk prediction.

Ceruloplasmin, an acute-phase protein, has been reported to increase in various acute inflammatory conditions, including injury, malignancy, cardiovascular disease, and infection [10], [11]. Additionally, an elevated level of ceruloplasmin has been closely correlated with some auto-immune diseases, such as rheumatoid arthritis and systemic lupus erythematous [18], [19]. Therefore, it is reasonable that ceruloplasmin may play an important role in aGvHD, which is also associated with immunological reactions and inflammatory cytokine storm. Because the oxidative status of blood and epithelial cells plays a critical role in the pathogenesis of aGvHD [20], and because ceruloplasmin is an antioxidant, this protein could be unregulated in oxidative stress with response to stimulating cytokines signaling [21]. Variations in ceruloplasmin levels during the pathogenesis of aGvHD may contribute to systemic negative feedback in vivo, which could assist in the regulation of cytokine storm and tissue damage during the anti-host attack. In addition, ceruloplasmin has also been shown to exhibit glutathione-peroxidase and nitric oxide-oxidase/S-nitrosating activities, which provide further cytoprotection [22], [23].

In kinetic studies of ceruloplasmin, patients with diagnosed infections at fixed time points were initially excluded to avoid potential interference. The myeloablative conditioning regimen appeared to promote increases in ceruloplasmin levels, which may be similar to the physiological changes in solid tumors during radiotherapy [24]. However, following graft infusion, the ceruloplasmin levels continued to increase in the aGvHD+ group but remained relatively stable in the aGvHD- group. Although it is premature to discuss whether the differences between the groups could be attributed to distinctive tissue injuries during conditioning or to graft composition, the ceruloplasmin kinetics provided clues for further research on the diagnostic and prognostic value of this biomarker.

Infections were one of the most common inflammatory complications after HSCT and may have interfered with the specificity of aGvHD biomarkers [4], [25]. Notably, our previously reported biomarker, LBP, also failed to differentiate aGvHD from infection [8]. While ceruloplasmin was previously reported to increase in infections [11], [26], [27], and the levels at diagnosis were similar to our results; yet there was no difference between the onset and recovery of infections in our allo-HSCT cohort, considering the relatively high baseline level of ceruloplasmin after conditioning regimen. On the other hand, we found that ceruloplasmin might have a better diagnostic value for aGvHD independent of infection status, as the elevation of ceruloplasmin at onset of aGvHD would be more remarkable than infections. Although the diagnostic time points of aGvHD and infections were not all the same, the disparity of ceruloplasmin kinetics between patients only with aGvHD and infection at multi-time points might contribute to explain this question. In addition, ceruloplasmin was found to be similar in aGvHD patients with or without infection. Because of this characteristic, ceruloplasmin showed potential better diagnostic specificity than LBP (AUC 0.861 Vs. 0.747).

Ceruloplasmin lacks organ specificity for skin- or GI-type aGvHD; this may be because ceruloplasmin levels rise in response to systemic conditioning rather than specific tissue damage. For instance, a validated skin-specific biomarker, elafin, is expressed by target keratinocytes rather than by the effector cells that are capable of damaging all three target organs [28]. A combination of systemic biomarkers and organ-specific markers would further improve the sensitivity and specificity of ceruloplasmin, especially in the differential diagnosis of many ambiguous cases [25]. Also elevation of ceruloplasmin were not related to the types of allo-HSCT, which suggested that ceruloplasmin might not rise corresponding to the specific conditioning regimens or donor sources. Although there was a tendency for the level of ceruloplasmin to be correlated with the severity of aGvHD, mixed-type and grade 2 aGvHD cases appeared to have higher ceruloplasmin levels at diagnosis. Indeed, we observed that patients’ ceruloplasmin levels continued to increase when their symptoms did not remit or worsened. In future studies, it will be useful to test the relationships between ceruloplasmin and both aGvHD stage and the probability of therapy response in a larger cohort.

Ceruloplasmin may have value not only as a noninvasive diagnostic biomarker but also as a prognostic factor for aGvHD pathogenesis. By periodically monitoring the ceruloplasmin levels at d+7, d+14 and d+21, patients who were at a higher risk of developing aGvHD could be screened before symptoms arose. Thus, risk-adapted prophylaxis for aGvHD has been shown to be effective in clinic (6), and it will be interesting to evaluate the role of ceruloplasmin in future preemptive treatment strategies.

In conclusion, the plasma ceruloplasmin level could be used as a potential biomarker for aGvHD diagnosis and for the prognosis of aGvHD onset at d+7, d+14, and d+21 after HSCT. We recommend that ceruloplasmin be combined with other validated biomarkers in a screening panel for aGvHD diagnosis and prophylaxis in future prospective, interventional clinical studies.

## Supporting Information

Figure S1 **Patients enrolled in this study.**
(TIF)

Figure S2 **Ceruloplasmin levels in aGvHD+ patients with or without infection.** Ceruloplasmin level in patients with(infection+) Vs. without infections (infection-)suffering grade-1 aGvHD (n = 16 Vs. n = 20, p = 0.972), grade-2 aGvHD (n = 15 Vs. n = 17, p = 0.436), while all patients with grade 3–4 aGvHD (n = 4) got infections.
(TIF)

Figure S3 **Comparison of ceruloplasmin in patients following HSCT from MRD(n = 20) and Haplo donors (n = 43) in systemic following-up.** There were not significant differences between these two groups (MRD Vs Haplo) in ceruloplasmin levels.
(TIF)

Table S1 (DOC)
