# Supplementary figures and images for: Ceruloplasmin Is a Potential Biomarker for aGvHD following Allogeneic Hematopoietic Stem Cell Transplantation
Source: PLoS One. 2013 Mar 7;8(3):e58735. doi: 10.1371/journal.pone.0058735 (PMC3591372; doi:10.1371/journal.pone.0058735)

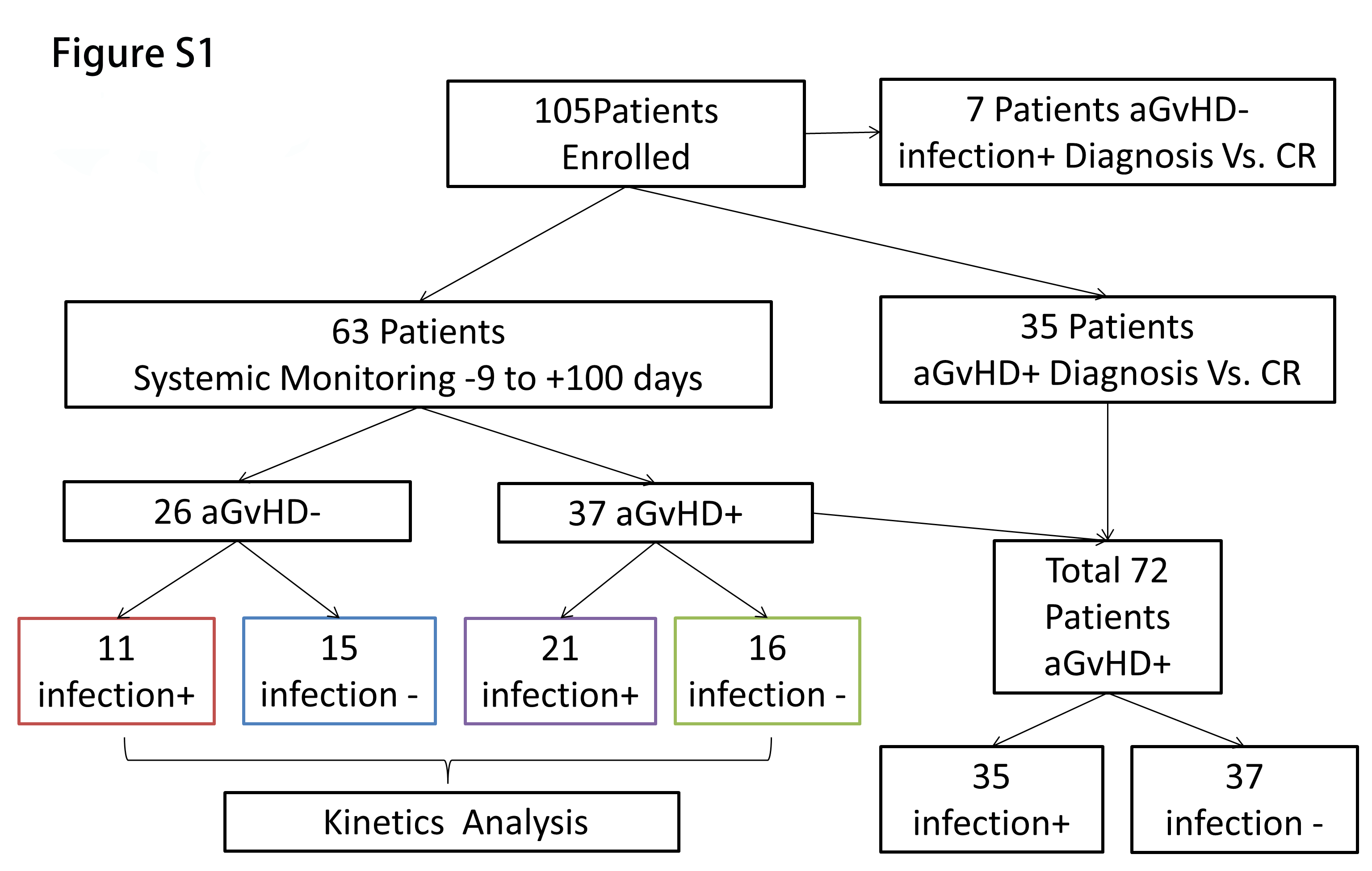

Supplement: Figure S1 — Patients enrolled in this study. (TIF) [file pone.0058735.s001.tif]

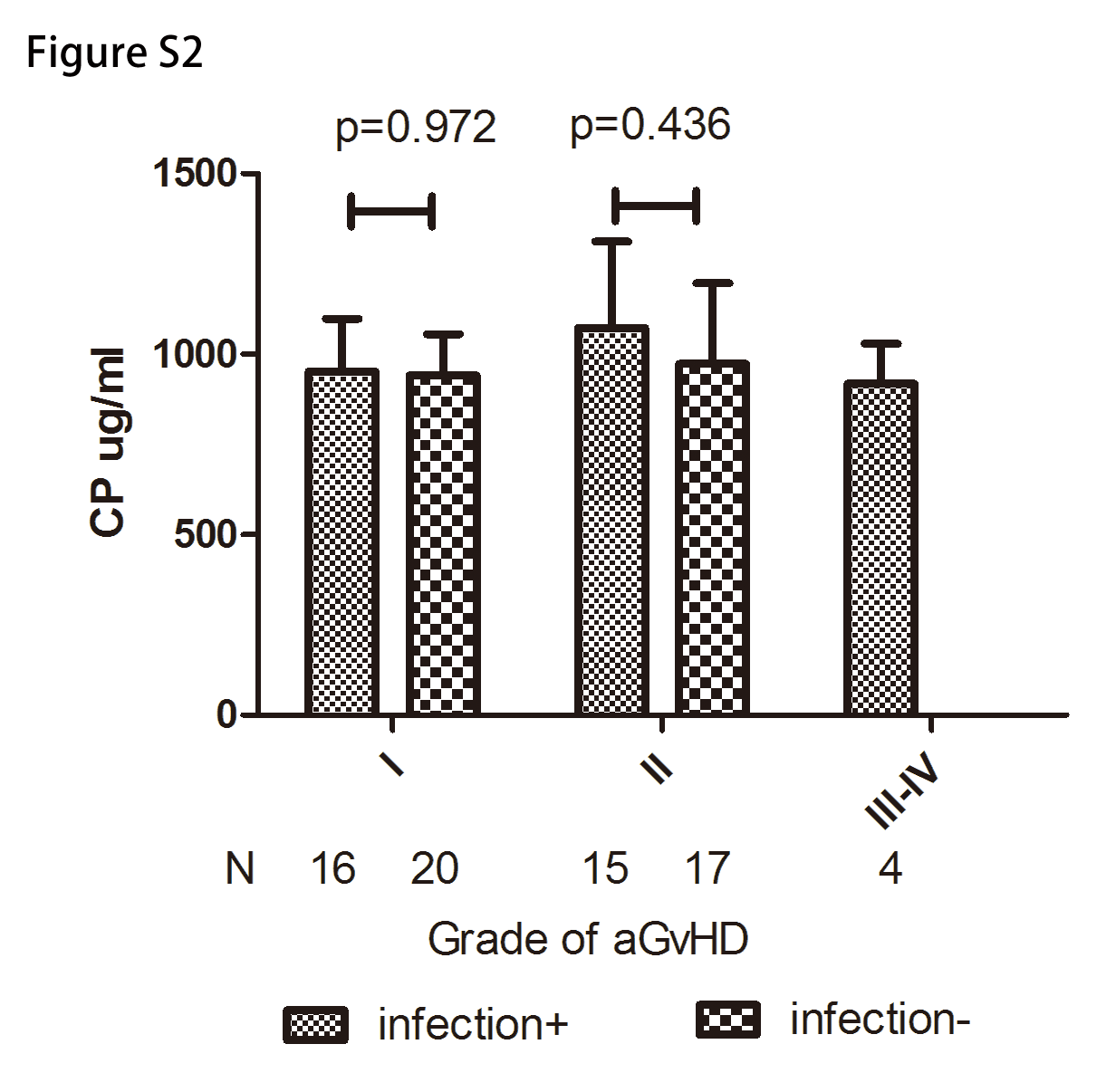

Supplement: Figure S2 — Ceruloplasmin levels in aGvHD+ patients with or without infection. Ceruloplasmin level in patients with(infection+) Vs. without infections (infection-)suffering grade-1 aGvHD (n = 16 Vs. n = 20, p = 0.972), grade-2 aGvHD (n = 15 Vs. n = 17, p = 0.436), while all patients with grade 3–4 aGvHD (n = 4) got infections. (TIF) [file pone.0058735.s002.tif]

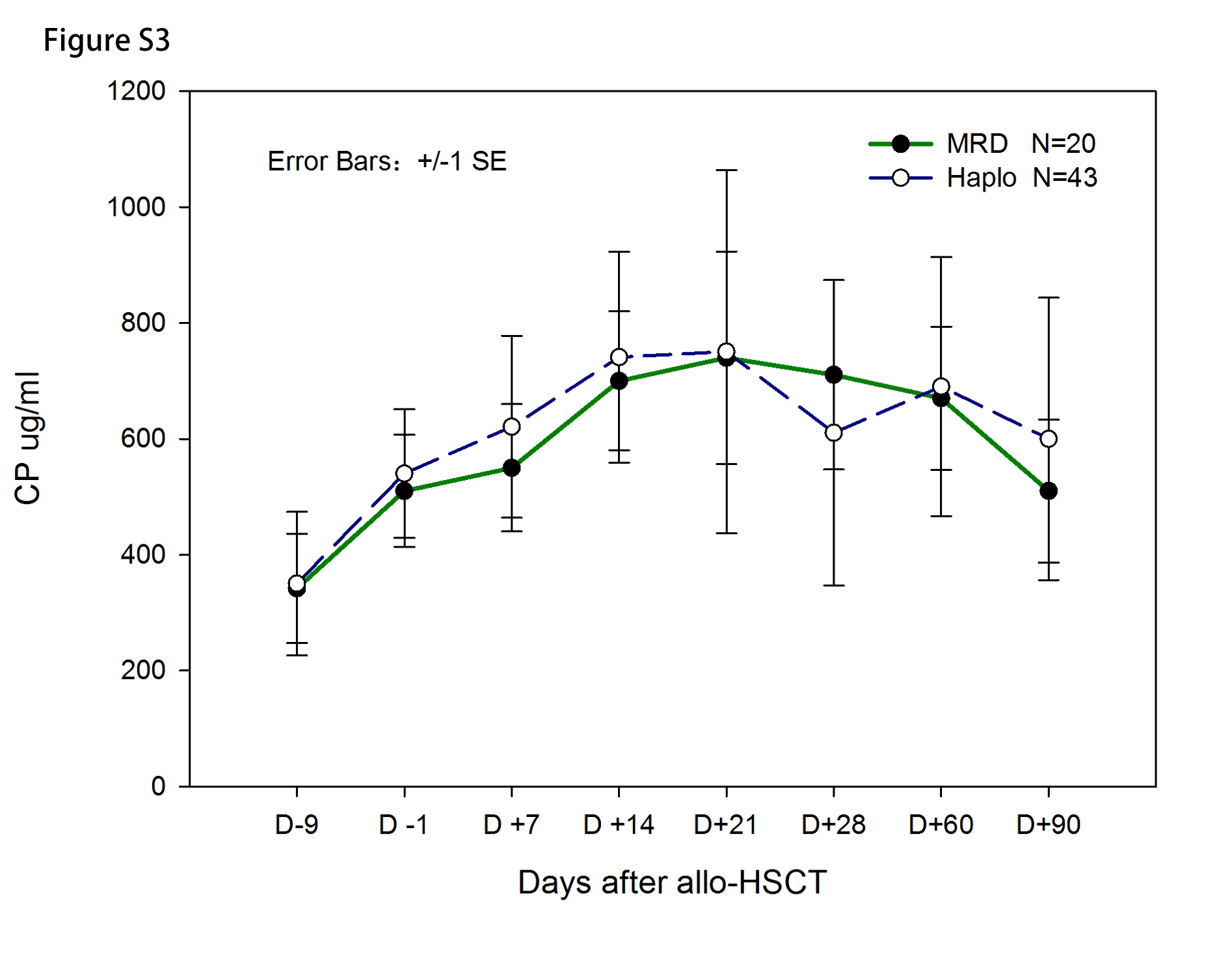

Supplement: Figure S3 — Comparison of ceruloplasmin in patients following HSCT from MRD(n = 20) and Haplo donors (n = 43) in systemic following-up. There were not significant differences between these two groups (MRD Vs Haplo) in ceruloplasmin levels. (TIF) [file pone.0058735.s003.tif]
