# Supplementary material for: Ceruloplasmin Is a Potential Biomarker for aGvHD following Allogeneic Hematopoietic Stem Cell Transplantation
Source: PLoS One. 2013 Mar 7;8(3):e58735. doi: 10.1371/journal.pone.0058735 (PMC3591372; doi:10.1371/journal.pone.0058735)
Supplement: Table S1 — (DOC) [file pone.0058735.s004.doc]

**Table S1**

Proteins with increased levels in aGVHD patients

| Up-regulated proteins | 113/114 | 115/116 | 117/118 | 119/121 |
| --- | --- | --- | --- | --- |
| Complement factor H | 1.5 | 1.3 | 1.9 | 1.8 |
| Myeloperoxidase | 3.2 | 2.9 | 2.2 | 1.7 |
| Ceruloplasmin | 2.1 | 2.9 | 1.3 | 2.2 |
| Alpha-1 acid glycoprotein | 1.6 | 2.4 | 2.2 | 1.5 |

Differential protein levels between aGVHD+ and aGVHD^-^ from four patients were evaluated for 113/114, 115/116, 117/118 and 119/121 sets, respectively, with the ratio more than 1.2 defined as increased.
